# Dr. John W. Francis

**Published:** 1861-03

**Authors:** 


### NECROLOGICAL NOTICES.

Dr. John W. Francis.—It is not the medical world alone which mourns the death of Dr. Francis, which took place on the eighth of February. So varied were his attainments and so versatile his genius, that his loss will fall with equal severity upon the social and literary, as well as medical circles. No one who knew him can ever forget the gentle, courteous manners and genial intercourse which characterized his daily demeanor during a life of seventy-two years.

Dr. Francis was born in New York City, on the 17th of November, 1789, of German and Swiss parentage. He entered Columbia College in 1807, and at the same time commenced his medical studies under Dr. Hossack, whose partner he subsequently became. He was graduated in 1811 by the College of Physicians and Surgeons, being, as we are informed, the first person upon whom a degree was conferred by that institution. His career as a practitioner and lecturer was eminently successful. No one was better calculated to enchain the attention of his audience, both from the elegant fluency of his diction and the many anecdotes with which he interspersed his discourse. He possessed a lively and keen sense of the humorous, and his fund of wit seemed of Protean fertility, always ready to burst forth, and charming every one with its prodigality of quaint conceits, and brilliancy of repartee.

As a physician he was greatly beloved, and the amount of gratuitous practice in which he engaged would seem incredible to those who, from personal experience, were not able to testify to the untiring charity and benevolence which guided his heart through life. The poor and afflicted were always sure of meeting with a warm response to their appeals, and a hearty endeavor to do his uttermost in alleviating their sufferings. But it was not only by the ignorant and sick that his influence was acknowledged and blessed. Many an author, toiling arduously for his subsistence, or devoting every energy to win the world's coveted plaudits, would have sunk disheartened under countless failures or scathing criticism, but for words of kind encouragement or seasonable assistance of a pecuniary nature bestowed by Dr. Francis. Every case of suffering appealed to his sympathy, and no thought of self ever for a moment intruded if he knew that his presence would bring the least relief to the sick or soothe the dying pillow of the poorest outcast. He would leave alike his own bright fireside and the most brilliant entertainment to mingle in such scenes.

Although highly susceptible to the ludicrous, Dr. Francis was naturally of a thoughtful disposition, and this trait was much increased by the death of a talented son in 1855. To this son he had always looked as a co-laborer in his professional exertions, hoping through his assistance to be in a great measure relieved from the cares and responsibilities to which his popularity and large practice subjected him.

Though the loss of this son cast a gloom over the remaining years of his life, he was especially blessed in his surviving children, and we feel justified in saying that no household ever boasted a more attractive or affectionate domestic circle. Not specifying it by any means as an isolated case, we can particularly understand how delightful was the home of Dr. Francis, not only from the many charming qualities of its inmates, but also from its being the resort of many of the leading authors in American literature and men celebrated in every department of science. His social circle comprised such men as Daniel Webster, America's greatest orator; Washington Irving, whose graceful pen has brought the Alhambra so vividly before us; Leslie, the gifted pupil of West, and most successful of all Shakspeare's delineators; Fennimore Cooper, the immortalizer of aboriginal America; Philip Freneau, the revolutionary poet; Richard H. Dana, Jared Sparks, Robert Walsh, and others.

It was when surrounded by men such as these, whose tastes were so congenial to his own, that Dr. Francis was in his element; nor was the part he sustained in their midst in the least subordinate. The wondrous depths of his intellect, his personal reminiscences of celebrated men both at home and abroad, the many anecdotes with which his memory was stored, and the saliency and keenness of his remarks, won for him golden opinions from men whose friendship was an honor, and served to give him prominency among the literati.

Dr. Francis was a prolific writer on promiscuous as well as medical subjects, and an interesting summary of the events of his life may be found in his well-known "Old New York, or Reminiscences of the Past Sixty Years." He contributed largely to various newspapers and magazines, wrote for Rees's Cyclopedia when abroad, and will long be remembered for the clearness and excellence of his medical writings.

Among his latest literary productions was the contribution of the lives of Dr. Macneven and Dr. Mitchill, to the American Medical Biography, by the senior editor of this Review. The dedication of it to Dr. Francis, as a "representative physician of an age now rapidly passing away," has been, alas, but too prophetic!

In 1813 the chair of Materia Medica was conferred upon him by his Alma

Mater, and a short time afterward he succeeded Dr. Stringham as Professor of Medical Jurisprudence, retaining this position until the dissolution of the faculty and the foundation, by a portion of it, of Rutger's Medical College, which, after a successful career of four years, was closed by the legislature. In 1847 he was elected the first President of the New York Academy of Medicine after its organization; and he was a member of many scientific institutions both abroad and at home. In 1850 he received the degree of LL.D. from Trinity College at Hartford.

After his retirement from public life as a lecturer, Dr. Francis devoted himself to a meritorious discharge of the duties of his profession, and to the pursuit of art and science under their most agreeable phases. He was an ardent devotee at music's shrine, and the exquisite compositions of Mozart, Rossini, and Donizetti always exercised a magical influence over him, utterly incapacitating him at the time for the exertion of any other faculty than the rapt sense of hearing.

After his return from Europe, which he visited in 1814, the society of Dr. Francis was eagerly sought and appreciated by the intellectual social cotéries which soon surrounded him. During his continental tour he was so fortunate as to meet with Abernethy and other eminent physicians, Sir Walter Scott, Burns, Kean, Tom Paine, Cuvier, Denon, Gall, Brewster, and many other men of similar renown.

Thanks to an excellent memory, he returned home with his mind well stored with interesting local and personal anecdotes and graphic accounts of contemporaneous history, whose recital was always a source of delight to his friends and admirers.

In bestowing upon him so many mental endowments, nature had not denied him the gift of physical attractions. We transcribe the following description from one of his numerous obituaries:—

"Though seventy-two years old, he was to all appearance hale and hearty, until a very recent date. His genial face, his curling white hair, the low hat and white cravat which he usually wore, the heavy gold-headed cane almost invariably within his grasp when out of doors, all combined to give him the appearance of the now legendary Knickerbocker."

Dr. Francis was a regular attendant at Calvary Church, and a warm personal friend of its rector, Dr. Hawks, by whom he was visited in his illness.

He retained his consciousness to the last, and immediately before expiring spoke earnestly of his faith in an all-merciful God, and his entire submission to his inscrutable will.

> "Nothing in his life
> Became him like the leaving it. He died
> As one that had been studied in his death
> To throw away the dearest thing he ow'd
> As 'twere a careless trifle."

